# Coupled capillary-cavitation model for secondary droplet breakup in high-intensity ultrasonic field

**DOI:** 10.1016/j.ultsonch.2026.108092

**Published:** 2026-10-01

**Authors:** Olga Kudryashova, Andrey Shalunov, Roman Dorovskikh, Sergey Titov, Natalia Titova

**Affiliations:** Biysk Technological Institute, Biysk, Russia

**Keywords:** Ultrasonic droplet breakup, Cavitation, Capillary waves, Rayleigh–Plesset equation, Parametric resonance, Secondary atomization, Acoustic radiation pressure

## Abstract

A coupled physics-based model is developed to describe secondary breakup of levitating droplets in a high-intensity ultrasonic standing wave field. The model uniquely integrates acoustic radiation pressure-induced droplet deformation, parametric excitation of capillary waves, radial oscillations and collective interaction of cavitation bubbles, and energy accumulation in the system. The methodology involves simultaneous numerical solution of the Mathieu–Hill equation, the Rayleigh–Plesset equation with inter-bubble pressure coupling, and energy conservation principles. A critical gas volume fraction α∼cr∼ ≈ 0.35–0.38 is identified as the bifurcation threshold between two distinct breakup regimes. Below this value, capillary-wave fragmentation dominates, yielding a fragmentation time of ∼250–300 ms and a minimum droplet size limited by the capillary wavelength (∼24.7 μm for water at 22 kHz). Above the threshold, collective bubble collapse prevails, reducing fragmentation time to ∼10–15 ms and producing droplets an order of magnitude smaller (∼1 μm), as predicted by a Weber-criterion formulation incorporating bubble wall collapse velocity. Analytical expressions for threshold acoustic pressures required to achieve minimum droplet size are derived and validated numerically. For thin disk-shaped droplets (kh << 1, relevant for droplets < 0.5 mm), the critical excitation pressure is frequency-independent and can reach 84 kPa, whereas for larger droplets it ranges between 15–19 kPa at 22–44 kHz. Predicted final droplet radii for water (28 μm), glycerol (22 μm), and ethanol (18 μm) at 35 kPa and 22 kHz agree quantitatively with experimental data. The proposed model offers a predictive framework for controlling aerosol dispersity by tailoring gas content, initial droplet size, and acoustic field parameters, thereby enabling energy-efficient, contactless atomization technologies with tunable droplet characteristics.

## Graphical abstract

**Figure:**
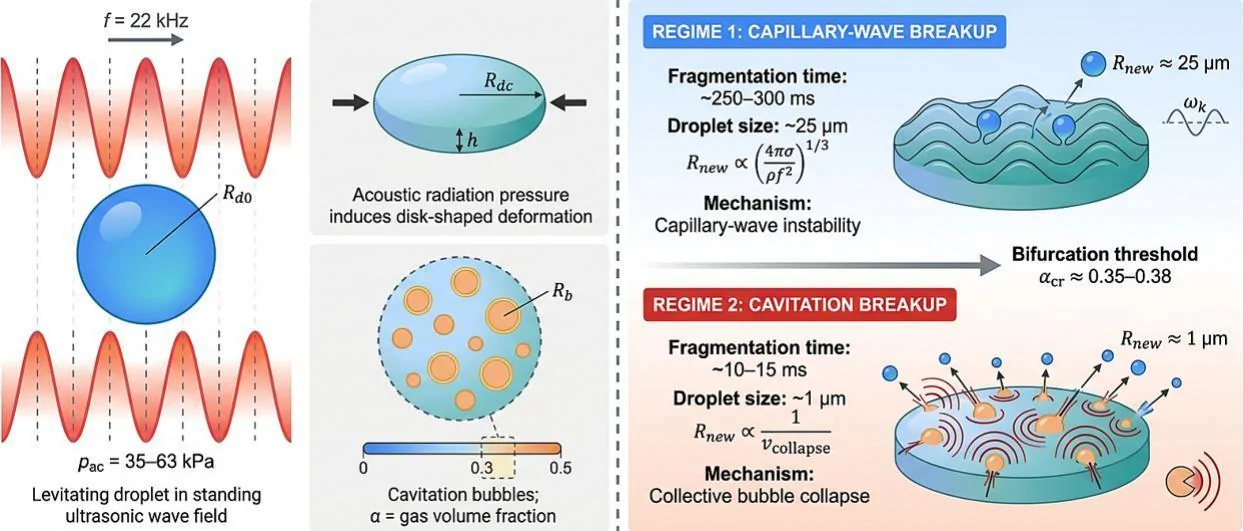

## Nomenclature

*A_uz_*: Relative modulation amplitude from ultrasonic action (dimensionless)
*A_b_*: Capillary wave excitation parameter by bubble pressure (dimensionless)
*c_g_*: Speed of sound in the gas (m/s)
*E_drop_*: Surface energy of the deformed disk-shaped droplet (J)
*E_new_*: Total surface energy of the newly formed droplets (J)
*E_uz_*: Energy accumulated by the system (bubbles and surface) during the induction time (J)
*f*: Ultrasound frequency (Hz (s^−1^))
*h*: Thickness of the deformed disk-shaped droplet (m)
*k*: Capillary wave number (m^−1^)
*k_B_*: Boltzmann constant (J/K)
*k_T_*: Pressure transmission coefficient into the droplet (dimensionless)
*l*: Characteristic size of the forming droplet (m)
*n_b_*: Number of cavitation bubbles in the droplet (dimensionless)
*n_d_*: Number of new droplets formed upon breakup (dimensionless)
*p*_0_: External (atmospheric) pressure (Pa)
*p_ac_*: Acoustic pressure amplitude (Pa)
*p_b_*: Pressure generated by neighboring cavitation bubbles (Pa)
*p_d_*: Pressure transmitted into the droplet (Pa)
*p_rad_*: Acoustic radiation pressure (Pa)
*p_s_*: Saturated vapor pressure (Pa)
*r*: Maximum distance over which the bubble influence is significant (m)
*t_c_*: Fragmentation (induction) time (s)
*v*: Characteristic velocity of the liquid in the wave crest (m/s)
*a_k_*: Capillary wave amplitude (m)
*R_b_*: Cavitation bubble radius (m)
*R_b_*_0_: Initial radius of the cavitation nucleus (m)
*R_d_*: Radius of the initial spherical droplet (m)
*R_d_*_0_: Initial radius of the original spherical droplet (m)
*R_dc_*: Radius of the deformed disk-shaped droplet (m)
*R_new_*: Radius of a new droplet formed upon breakup (m)
*T*: Temperature (K)
We: Weber number (dimensionless)
α: Gas (bubble) volume fraction in the liquid (dimensionless)
α*_cr_*: Critical gas volume fraction for the transition between regimes (dimensionless)
γ: Adiabatic index of the vapor in the bubble (dimensionless)
η: Dynamic viscosity of the liquid (Pa·s)
ν: Kinematic viscosity of the liquid (m^2^/s)
ξ: Surface disturbance amplitude (wave height) (m)
ξ*_cr_*: Critical wave amplitude at which droplet detachment occurs (m)
ρ: Liquid density (kg/m^3^)
ρ*_g_*: Gas density (kg/m^3^)
σ: Liquid surface tension (N/m)
ω: Angular frequency of ultrasound (rad/s)
ω*_k_*: Natural frequency of the capillary wave (rad/s)
μ: Exponential growth increment of the capillary wave (s^−1^)

## 1 Introduction

The fragmentation of liquid droplets under the influence of an ultrasonic field is a complex, multifactorial process involving the interaction of acoustic, hydrodynamic, and capillary effects. Of particular interest is the secondary fragmentation of levitating droplets in the standing wave node of an ultrasonic field, since in this case there is no mechanical contact with the surface, which is important for certain technological processes. The dynamics of a levitating droplet are determined solely by the structure of the acoustic field and the internal processes in the liquid. Such modes are used in contactless atomization technologies, the generation of highly dispersed aerosols, emulsification, ultrasonic cleaning, and biomedical and microfluidic applications. For example, in inhalation therapy, it is important to obtain droplets of 1–5 μm rather than 25 μm. Moreover, in many applications, the desired characteristic is the minimum droplet size and a narrow size distribution of the resulting droplets. This is important because finely dispersed droplets have a large specific surface area, which accelerates heat and mass transfer, increasing the efficiency of evaporation and chemical reactions. It is also important to create a fine aerosol with high performance. Under identical process conditions, a high aerosol dispersion allows for reduced substance consumption while maintaining effective action.

Despite a long history of research, beginning with the classic works of Wood and Loomis [1], and later Lang [2], Topp [3], and Danilov and Mironov [4], a unified theory of ultrasonic droplet fragmentation has yet to be developed. Modern research shows that droplet fragmentation can occur through several competing or cooperative mechanisms [4]: due to the parametric excitation of capillary waves, the action of acoustic radiation pressure, the cavitation collapse of bubbles, the formation of microjets, and nonlinear thermoacoustic effects.

The capillary-wave mechanism is considered one of the basic scenarios of ultrasonic atomization [5], [6], [7], [8], [9], [10], [11], [12]. According to this model, the acoustic impact excites standing capillary waves on the droplet surface. Upon reaching a critical amplitude, these waves become unstable, leading to the detachment of daughter droplets from the wave crests. The size of the resulting fragments is related to the capillary wave length, determined by the ultrasound frequency, the liquid density, and the surface tension. However, experiments show that the capillary-wave model alone is insufficient to describe secondary fragmentation at high ultrasound intensities [13].

At moderate ultrasonic intensities, droplet breakup can be described predominantly by the instability of acoustically excited capillary waves, with daughter droplets detaching from the wave crests. At higher intensities, however, cavitation becomes increasingly important. The nonlinear oscillations and subsequent collapse of cavitation bubbles generate localized pressure pulses, shock waves, and microjets, producing liquid velocities that can substantially exceed those associated with the growth of capillary-wave instability. Consequently, when bubble collapse becomes the dominant breakup mechanism, the characteristic droplet size is no longer determined solely by the capillary wavelength. In this regime, the capillary-wave description becomes incomplete because it does not account for the internal energy release and highly localized momentum transfer associated with cavitation collapse.

Another mechanism under consideration is cavitation breakup [3], [8], [9], [10]. Upon reaching the cavitation threshold, bubbles are formed in the liquid, whose oscillations and collapse are accompanied by localized shock waves, microjets, and intense pressure gradients. These effects can break apart the droplet from within. High-speed visualization and numerical modeling show that in powerful ultrasonic fields, bubble collapse significantly enhances liquid fragmentation [3]. A number of modern studies (including the authors' own) directly link secondary fragmentation to the accumulation of energy by cavitation bubbles and the subsequent catastrophic breakup of the droplet [11], [12], [13], [14].

However, modern experimental data show that the actual fragmentation process is not limited to the isolated action of a single mechanism. Before disintegration, a droplet typically undergoes a stage of strong deformation: under the influence of acoustic radiation pressure, it acquires a flattened or disc-shaped form, which significantly changes the local conditions of capillary wave excitation and the dynamics of cavitation bubbles [13], [15]. Furthermore, in intense acoustic fields, bubble interactions arise through secondary Bjerknes forces, bubble clusters and collective collapse modes are formed, which can radically change the local energy concentration. Seko et al. [16] hypothesized that oscillations of cavitation bubbles can play a significant role in the detachment of droplets from capillary wave crests, which links the two main mechanisms.

The method of cavitation-induced droplet fragmentation in ultrasonic fields proposed in [13] allowed for an experimental demonstration of the feasibility of secondary droplet fragmentation in a powerful ultrasonic field. At a sound pressure level of 35 kPa, the minimum droplet radii obtained during primary droplet fragmentation (∼1-5 mm) were 28 μm for water, 22 μm for glycerin, and 18 μm for alcohol. It was shown that two levels of critical sound pressure exist: the pressure limit at which droplet fragmentation occurs, and the saturation pressure, above which the reduction of the resulting droplets ceases. As demonstrated in this work, this result can only be explained by considering cavitation phenomena in the droplet.

In recent years, research has shifted from an empirical description of breakup thresholds to the construction of models linking the structure of the acoustic field, droplet deformation, the development of instabilities, and the resulting droplet dispersion [5], [14]. To find new modes for efficient droplet fragmentation, attention should be paid to multi-frequency and dual-frequency modes of action, the nonlinear spectral composition of excitation, and the influence of viscosity and rheological properties of the medium. For example, it has already been shown that viscosity can influence the breakup threshold more strongly than surface tension [3], [5], and non-sinusoidal excitation modes significantly alter bubble lifetime and fragmentation patterns [17].

Existing models consider capillary breakup or cavitation separately. However, most existing models focus either on a single breakup mechanism or on a narrow range of conditions: only Newtonian fluids, single-frequency sinusoidal excitation, and the absence of collective bubble dynamics or strong droplet deformation. This limits the applicability of existing approaches to secondary breakup control problems under complex impact conditions and when working with highly viscous liquids, non-Newtonian media, or suspensions.

Thus, a literature review reveals several significant limitations of current models of secondary ultrasonic droplet breakup. Most models consider individual breakup mechanisms in isolation: either cavitation, capillary-wave, or radiation deformation, whereas in real ultrasonic fields, these processes are interconnected. The influence of strong deformation of a levitating droplet and its transition to a disc-shaped form on the conditions for capillary wave excitation, acoustic pressure distribution, and cavitation bubble dynamics has been insufficiently studied. Collective effects of bubble interactions are poorly understood, although they may determine energy localization and initiation of decay.

The criteria for transitioning between different fragmentation mechanisms depending on field parameters and fluid properties are insufficiently developed, especially for highly viscous, viscoelastic, and non-Newtonian media, as well as suspensions. Meanwhile, the dominant mechanism determines the minimum achievable droplet size, the size distribution width, the energy efficiency of the process, and spray stability. The novelty of our work lies in the adjoint energy criterion, which allows us to predict which mechanism is dominant and how droplet size changes during the transition.

The transition between mechanisms often has a threshold nature and is accompanied by a sharp change in aerosol dispersion. Therefore, for engineering process control, it is necessary to know the transition criteria between modes, expressed in terms of acoustic field parameters, fluid properties, and cavitation characteristics. Only then can we design ultrasonic systems to achieve the desired dispersion, minimize energy consumption, and ensure process stability.

The aim of this work is to develop a model for the secondary fragmentation of levitating liquid droplets in an ultrasonic field. This model generally takes into account the accumulation of energy by cavitation bubbles; radiation-induced droplet deformation; the excitation and development of capillary waves on the droplet surface; the collective dynamics and interaction of cavitation bubbles; and the influence of viscosity, non-Newtonian properties, and the dispersed structure of the liquid. Based on this model, we intend to establish transition criteria between different fragmentation mechanisms and determine the conditions for the formation of the finest fragments under various acoustic exposure conditions.

In Section 1, we formulate the equations for deformation, capillary waves, and cavitation. In Section 2, we present numerical results and a parametric study. In conclusion, we discuss the transition criteria and the applicability of the model.

## 2 Statement of the problem. Basic equations

A liquid droplet levitates in a standing wave of a powerful ultrasonic field. When ultrasonic vibrations interact with the liquid droplet, various mechanisms occur within it, leading to a change in its shape and, under certain conditions, its disintegration into smaller droplets. The droplet is deformed into a disk shape under the action of radiation pressure generated by the sound wave, if this pressure is sufficient to overcome the droplet's surface tension. The variable sound pressure initiates vibrations of vapor-gas cavities within the droplet-cavitation bubbles (Fig. 1). Cavitation bubbles in the acoustic field will oscillate and possibly collapse, depending on the ultrasonic conditions. This differs from generating acoustic vibrations in a continuous liquid medium in that it is impossible to transmit high-power vibrations into the liquid through air due to significant vibration reflection at the droplet boundary. Furthermore, the droplet boundaries are mobile and can be deformed under the action of radiation pressure.

**Fig. 1:**
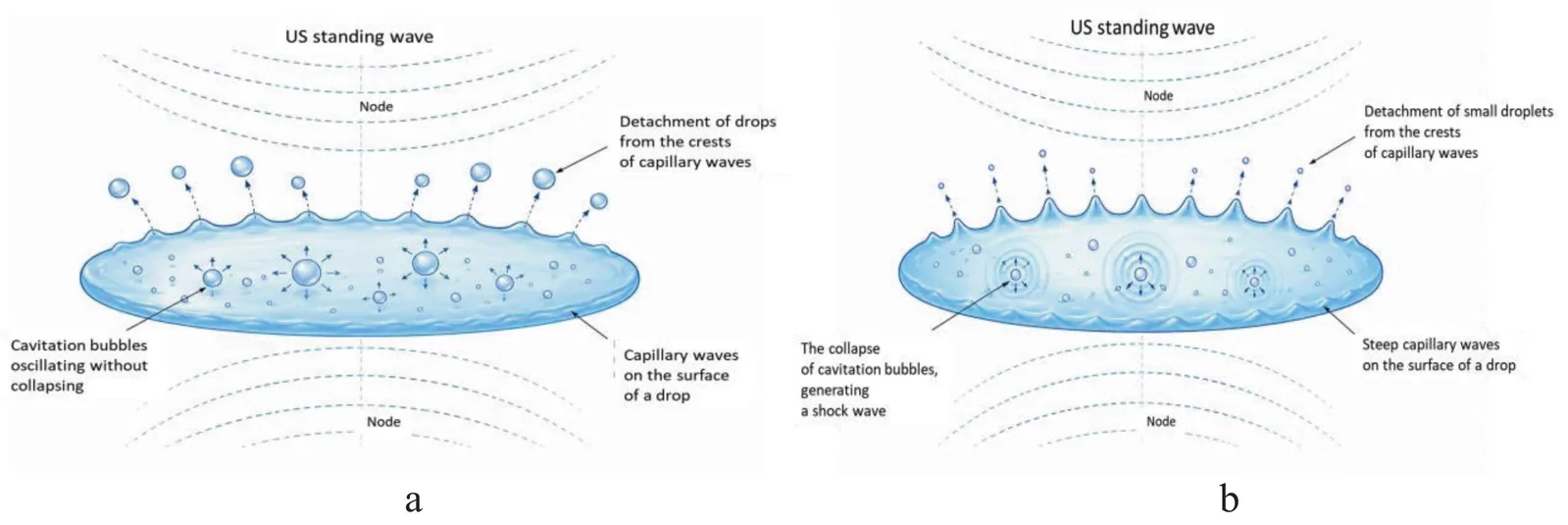
Schematic representation of the two main mechanisms of droplet destruction: capillary (a) and cavitation (b).

The mechanism of ultrasonic capillary dispersion of liquid from a layer is the detachment of new droplets from capillary wave crests (Fig. 1a). We will consider the finite thickness of the layer (disc-shaped droplet) and the energy accumulation by oscillating cavitation bubbles during the induction period. This approach will allow us to describe how the ultrasonic energy accumulated by the bubbles causes new droplets to emerge from the surface of the initial droplet.

We will also consider the mechanism of cavitation breakup of a droplet – when cavitation bubbles collapse and generate shock waves toward the droplet surface (Fig. 1b). In this case, the surface wave crests rapidly accelerate, and the droplets detaching from them are smaller than in the case of “quiet” capillary-wave breakup. We will determine the criteria for the transition from one breakup mechanism to another, the minimum droplet size, and the dispersion characteristics of the new droplets, depending on the ultrasonic parameters and the properties of the liquid.

We will consider the following assumptions.

1. It is assumed that an ensemble of cavitation bubbles of equal size forms in a suspended droplet under the influence of ultrasound. We will take into account the mutual influence of the bubbles on each other and on the droplet surface in the form of pressure pulses.
2. Under the influence of the radiation pressure of a standing ultrasonic wave, the droplet is deformed into a disk-shaped form. We will take into account that the bubble oscillations occur not in a spherical droplet, but within the deformed droplet (that is, in a flat liquid layer of a certain thickness).
3. The oscillations of the cavitation bubbles occur over a certain induction time, which is accompanied by the accumulation of ultrasound energy in the bubble. When the accumulated energy exceeds the surface tension energy of the droplet, the droplet breaks up into new fragments, the surface energy of which is determined by the ultrasound energy accumulated in the cavitation bubbles.

### 2.1 Droplet deformation in a standing ultrasonic wave node

In a standing ultrasonic wave, a drop of liquid of radius *R_d_*_0_ is deformed under the action of radiation pressure *p_rad_* over a certain time (induction) *t_ind_* into a disk-shaped form with radius *R_dc_* and thickness *h* (these parameters are obtained from the problem of deformation of a drop of radius *R_d_*_0_ in an ultrasonic field [13]):

$$R_{\mathrm{dc}}=R_{d0}+\frac{p_{\mathrm{rad}}R_{d0}^{2}}{\sigma },h=\frac{4}{3}\frac{R_{d0}^{3}}{R_{\mathrm{dc}}^{2}}.$$

$$R_{\mathrm{dc}}\left(t\right)=R_{d0}+\frac{p_{\mathrm{ad}}\cdot R_{d0}^{2}}{\sigma }\left(1-e^{-t/t_{\mathrm{ind}}}\right)$$

$$t_{\mathrm{ind}}∼\frac{\eta R_{d0}}{\sigma },$$

where η is the dynamic viscosity and σ is the surface tension of the liquid. Cavitation bubbles form and oscillate within the liquid. We assume that all bubbles have the same radius *R_b_*.

The deformation model used here was developed and experimentally validated in our previous study [13] using high-speed visualization of levitating droplets in a standing ultrasonic field. The experiments showed that the initially approximately spherical droplet progressively flattens under the action of acoustic radiation pressure, approaching a disk-like geometry. In the present model, this deformation is quantified by the time-dependent disk radius *R_dc_*(*t*) and thickness (*h*), rather than being prescribed as an instantaneous change in geometry. The resulting deformed geometry is subsequently used in the calculations of cavitation-bubble dynamics and surface-wave development. Thus, the cavitation bubbles are considered to oscillate within a finite-thickness liquid layer corresponding to the acoustically deformed droplet, providing a direct coupling between radiation-pressure-induced deformation and the subsequent fragmentation dynamics.

### 2.2 Cavitation inside a droplet and capillary waves on the droplet surface

When a drop is exposed to ultrasonic waves in a standing ultrasonic wave, the pressure on the surface of the drop can be written as follows:

(1) $$p_{\mathrm{ext}}\left(t\right)=p_{0}+p_{\mathrm{ac}}\cos\left(\omega t\right)+p_{\mathrm{rad}}+p_{b}\left(t\right),$$

where *p_ac_* is the amplitude of the acoustic pressure $p_{\mathrm{rad}}=\frac{p_{\mathrm{ac}}^{2}}{4\rho_{g}c_{g}^{2}}$, ω is the ultrasound frequency, *p*_0_ is the external pressure, *p_rad_* is the radiation pressure, ρ*_g_*, *c_g_* are the density and speed of sound in the gas, *p_b_* are the disturbances from collapsing bubbles.

In general, under the influence of sound pressure, cavitation develops inside a droplet, i.e., oscillations of cavitation bubbles occur. Under certain conditions, the collapse of vapor-gas bubbles occurs. It should be noted that typically a small portion of the ultrasound energy is transferred to the liquid. The transmission coefficient is considered to be 0.1%, and in gas-saturated liquids – 2-3% [27]. Thus, even if powerful ultrasound vibrations are generated in the air, a small portion of their energy will be transferred to the liquid, which may indicate the absence of developed cavitation with bubble collapse. However, some studies show that a concave liquid surface can significantly enhance the ultrasonic transmission efficiency; the reported enhancement can reach up to 80% relative to the flat-surface case [18]. A concave surface is formed if capillary waves arise on the liquid surface. Thus, as capillary waves develop on the surface, the proportion of ultrasound energy transferred from the air to the liquid increases (linearly with an increase in the capillary wavelength). We obtain a positive feedback system: as capillary waves develop, the transfer of ultrasonic energy into the droplet increases, which, in turn, intensifies cavitation processes. The developing cavitation delivers increasing pressure disturbances (resulting from the collapse of bubbles) to the droplet surface, which intensifies the capillary waves.

When ultrasound is applied to the surface of a liquid or the interface between two phases, standing capillary waves arise. The dynamics of disturbances on the surface ξ(t) are described by the Mathieu-Hill equation [8], [19], [20]:

(2) $$\frac{d^{2}\xi }{dt^{2}}+2\gamma_{v}\frac{d\xi }{\mathrm{dt}}+\omega_{k}^{2}\left(1+A_{\;\mathrm{mod}\;}\cos\left(\omega t\right)\right)\xi =0,$$

where ω_k_ is the natural frequency of the capillary wave, $\omega_{k}^{2}=\frac{\sigma }{\rho }k^{3}$ [21]. $A_{\;\mathrm{mod}\;}\approx A_{\mathrm{uz}}=\frac{p_{\mathrm{ac}}}{\rho_{g}c_{g}^{2}},$*A*_uz_ is the relative amplitude of modulation from ultrasonic action, ω is the ultrasound frequency, γ*_v_* is the viscous attenuation $\gamma_{v}=2\upsilon k^{2},$ υ is the kinematic viscosity, and *k* is the wave number. The initial condition for equation (2) is determined from the assumption of thermal fluctuation as the initial disturbance on the surface: $\xi \left(0\right)\approx \sqrt{\frac{k_{B}T}{\sigma k^{2}}},$$\dot{\xi }\left(0\right)=\omega_{k}\xi \left(0\right),$ where *k_B_* is the Boltzmann constant, and *T* is the temperature.

The resulting waves are subharmonic, meaning they oscillate at exactly half the ultrasound frequency, with ω ≈ 2ω*k*. When the ultrasound pressure amplitude exceeds a certain threshold, parametric resonance occurs, leading to an exponential increase in the wave amplitude and subsequent droplet breakup. Equation (2) does not include the contribution of cavitation. We account for the effect of pressure pulses from cavitation bubbles by adding to the amplitude of capillary wave modulation due to ultrasound:

(3) $$A_{\;\mathrm{mod}\;}\approx A_{\mathrm{uz}}+A_{b},$$

where $A_{b}=\frac{p_{b}}{\sigma k},$ *p_b_* is the pressure generated by the bubbles closest to the surface. Thus, the pressure from the bubbles modulates the effective amplitude of the ultrasonic impact on the surface. The number of bubbles *n_b_* is a parameter dependent on the gas saturation of the liquid. The concentration of bubbles (cavitation nuclei) can be of the order of ∼106 – 108 1/cm3. [27] The volume fraction of gas in the liquid is α. Then the number of bubbles in a drop is $n_{b}=\alpha {\left(\frac{R_{d0}}{R_{0}}\right)}^{3},$

(4) $$p_{b}∼\rho n_{b}\frac{R_{0}}{r}R_{b}\frac{dR_{b}^{2}}{dt^{2}}.p_{b}∼\rho n_{b}\frac{R_{0}}{r}R_{b}\frac{dR_{b}^{2}}{dt^{2}}.$$

This pressure is proportional to the bubble size, the acceleration of its surface oscillations, the liquid density, and the number of bubbles nb. The bubbles exert the same pressure on each other. Equation (4) also includes the maximum distance r, at which the influence of bubble pressure on the surface or other bubbles is significant. The ratio of the initial bubble radius to this distance can be expressed through the volume fraction of bubbles:$k_{b}=\frac{R_{0}}{r}=\sqrt[{3}]{\frac{3\alpha }{4\pi }}.$

The radial oscillations of a cavitation bubble are described by the Rayleigh-Presset equation [22], [23]:

(5) $$R_{b}\frac{d^{2}R_{b}}{dt^{2}}+\frac{3}{2}{\left(\frac{dR_{b}}{\mathrm{dt}}\right)}^{2}+\frac{1}{\rho }\left[p_{0}-p_{s}+p_{b}-p_{d}\sin\left(\omega t\right)+\frac{2\sigma }{R_{b}}+\frac{4\eta \;}{R_{b}}\frac{dR_{b}}{\mathrm{dt}}-\left(P_{0}+\frac{2\sigma }{R_{0}}\right){\left(\frac{R_{0}}{R_{b}}\right)}^{3\gamma }\right]=0.$$

Here *p_s_* is the saturated vapor pressure, η is the dynamic viscosity of the liquid, *p*_0_ is the external (atmospheric) pressure, γ is the adiabatic index of the vapor in the bubble, σ is the surface tension of the liquid, ρ is its density, *p_d_* = *p_ac_ k_T_*, *k_T_* is the coefficient of pressure transmission into the drop. According to [18], this coefficient increases linearly with increasing wave height on the surface ξ: *k_T_* = 0.01+ *k_T_*_1_ ξ. *k_T_*_1_ ∼ 270 1/m (according to [18]). Thus,

(6) $$p_{d}=p_{\mathrm{ac}}\left(0.01+k_{T1}\xi \right).$$

The coefficient *k_T_* represents the pressure transmission into the droplet rather than the fraction of the incident acoustic energy transmitted through the interface. Its dependence on the surface-wave amplitude, *k_T_* = 0.01+ *k_T_*_1_ ξ, describes the experimentally established enhancement of acoustic-pressure transfer associated with the deformation of the liquid surface [18]. As the initially approximately flat surface develops a finite curvature due to capillary-wave deformation, the local acoustic interaction with the liquid interface changes, resulting in an increase in the pressure transmitted into the droplet. Therefore, the effect of surface concavity is expressed in the model as a relative enhancement of pressure transmission, while the absolute value of *k_T_* remains small.

For the conditions considered in the present study, the undeformed-surface value is *k_T_* =0.01. At the critical capillary-wave amplitude ξ =25 μm, the model gives *k_T_* = 0.017. Thus, the pressure-transmission coefficient increases by approximately 67.5% relative to the undeformed surface. This relative enhancement should not be interpreted as an absolute acoustic-energy transmission of 67.5–80%. The relation used for *k_T_* is applied within the range of surface deformations considered in the present model and should not be regarded as a universal relation for arbitrary acoustic conditions or droplet geometries.

The initial condition for equation (5): *R*_b_(0) = *R*_0_ (the initial radius of the cavitation nucleus). To account for the mutual influence of bubbles, a local pressure pb generated by surrounding cavitation bubbles (4) is introduced. By solving equations (2-6) together, we obtain the simultaneous dynamics of surface waves and cavitation bubbles.

The main objective of solving the equations of bubble oscillations (5) and cavitation waves (2) is to find the conditions for loss of stability (droplet breakup): bubble collapse or exponential growth of a capillary wave. This will also allow us to determine the time of droplet breakup and the sizes of new droplets. The stability zones for capillary waves in the simple case of harmonic oscillations and without taking into account the influence of cavitation bubbles are known. For the Mathieu equation, the threshold for loss of stability of capillary waves is known in the form [20]:

(7) $$A_{\;\mathrm{mod}\;}>A_{\mathrm{cr}}=\frac{2\gamma_{v}}{\omega_{k}},$$

Then $A_{\mathrm{cr}}=4\upsilon \sqrt{\frac{\rho k}{\sigma }},$ and the threshold acoustic pressure is $p_{\mathrm{acr}}=\sigma kA_{\mathrm{cr}}\approx 4\upsilon k^{3/2}\sqrt{\rho \sigma }.$

For a thin disk of thickness *h*, into which a droplet is deformed under the action of radiation pressure, the destruction mechanism changes. The natural frequency of a capillary wave in a thin layer is: The new threshold for a thin layer (*kh* << 1) is determined as $\omega_{k}^{2}=\frac{\sigma }{\rho }k^{3}\tanh\left(kh\right)\approx \frac{\sigma }{\rho }k^{4}h.$

### 2.3 Droplet destruction criterion and assessment of the size of new drops (excluding cavitation)

From the formulas given, we can analytically determine the droplet breakup thresholds under the influence of harmonic ultrasonic vibrations. In a simplified formulation, without taking into account the mutual reinforcement of capillary and cavitation processes, we obtain the size of new droplets. The size of a detached droplet is determined by the balance of forces at the moment of detachment. A droplet detaches when the kinetic energy of local motion exceeds the surface tension energy holding the liquid. This means that the droplet scale is determined by local wave parameters, not by the entire droplet. The detachment criterion is determined by the Weber number, which in this problem is expressed as follows [24]:

(8) $$\text{We =}\;\frac{\rho v^{2}l}{\sigma }>1,$$

where *v* is the characteristic velocity of the liquid in the crest, *l* is the characteristic size of the forming droplet (or the radius of curvature of the crest). The vertical velocity of the liquid in the wave is defined as *v* = *a_k_* ω*_k_*, where *a_k_* is the amplitude of the capillary wave. Then the droplet size (at We = 1) is determined as follows: $\xi_{\mathrm{cr}}∼\frac{\sigma }{\rho v^{2}}=\frac{1}{k^{3}a_{k}^{2}}$. The detachment of a new droplet is possible if the crest becomes sufficiently sharp, i.e., *k a_k_* ∼ *O*(1), $\xi_{\mathrm{cr}}∼\frac{1}{k}.$ That is, the size of the new droplet is inversely proportional to the wave number of the capillary wave (and is of the order of the capillary wave length). Then, knowing the relationship between the natural frequency of the capillary wave and the wave number – $k={\left(\frac{\omega_{k}^{2}\rho }{\sigma }\right)}^{1/3}$– we obtain the well-known formula for the droplet size for ultrasonic atomization [25]:

(9) $$R_{\mathrm{new}}∼{\left(\frac{\sigma }{\rho \omega_{k}^{2}}\right)}^{1/3}.$$

We will use this formula as a first approximation for estimating the size of new droplets. A more accurate result will be obtained from a numerical solution of the system of equations (2-6) taking into account criterion (8), where the velocity *v* = dξ/d*t*, ξ = ξ_cr_.

In the numerical solution, the dependence of dynamic viscosity on shear rate (for highly viscous and non-Newtonian fluids) can be considered: η = *f*(dξ/dt).

### 2.4 Time of capillary resistance violation

Capillary instability is an exponential growth of the leading mode of oscillation: $\xi \left(t\right)=\xi \left(0\right)e^{-\mu t}.$ Instability occurs when the amplitude reaches a critical level: ξ(*t_c_*) = ξ_cr_. Then $t_{c}=\frac{1}{\mu }\ln\frac{\xi_{\mathrm{cr}}}{\xi (0)}.$ The critical amplitude will be proportional to the wavelength (as shown above), $\xi_{\mathrm{cr}}∼\frac{1}{k}.$ Thus, the capillary fragmentation time can be estimated as:

(10) $$t_{c}=\frac{1}{\mu }\ln\frac{1}{k\cdot \xi (0)}.$$

For parametric excitation of capillary waves (the widest instability zone): $\mu \approx \frac{\omega_{k}}{4}\left(\frac{A_{\;\mathrm{mod}\;}}{A_{\mathrm{cr}}}-1\right),$$A_{\mathrm{cr}}=4\nu \sqrt{\frac{\rho k}{\sigma }},$ or for a thin layer $A_{\mathrm{cr}}\approx 4\nu \sqrt{\frac{\rho }{\sigma h}},$$A_{\;\mathrm{mod}\;}\approx A_{\mathrm{uz}}=\frac{p_{\mathrm{ac}}}{\rho_{g}c_{g}^{2}}$. Typical growth increment values for water are µ ∼ 100-1000 s^-1^.

Depending on the conditions, different droplet breakup scenarios and mechanisms can occur. These mechanisms are discussed below.

### 2.5 The size of new droplets taking into account the developed cavitation inside the original droplet

So far (in 2.3, 2.4), we have considered the case of capillary rupture of the droplet surface, where the fluid is not the determining factor. What is important is the detachment of new droplets as a result of the instability of capillary waves on the surface. In this case, the bubbles within the droplet oscillate without collapsing and without directly affecting the surface.

However, a situation can arise where the bubbles collapse, generating shock waves within the liquid, or have large radial oscillation amplitudes comparable to the layer thickness. We will call this situation developed cavitation. Then, near the surface, pulsed pressures, microjets, and local accelerations arise, significantly exceeding the surface acceleration of the capillary wave. The detachment of droplets from the surface of the deformed droplet ceases to be a wave-like process and becomes a localized shock-like process. The transition to developed cavitation is determined by solving the Rayleigh-Plesset equation (5). The Weber criterion (8) still determines surface rupture and the formation of new droplets. However, the velocity v in this case is determined by the local velocity of the liquid induced by the cavitation bubble. From (8) it follows that the radius of the new droplets is determined by:

(11) $$R_{\mathrm{new}}\approx \frac{\sigma }{\rho v^{2}}.$$

The calculation of the Weber criterion takes into account the sum of the velocities $v=\frac{d\xi }{\mathrm{dt}}+\frac{dR_{b}}{\mathrm{dt}}$ – the vertical velocity of the capillary wave crest and the velocity of the wall of a bubble located close to the surface. For a cavitation bubble of radius *R_b_*, collapsing with a wall velocity of d*R_b_*/d*t*, located at a distance of the order of its radius from the surface, the velocity *v* ∼ d*R_b_*/d*t*. This velocity is higher than the vertical velocity of the liquid at the capillary wave crest and determines the size of the new droplets (which will be smaller than with a “quiet” capillary-wave mechanism of surface destruction).

The local liquid velocity induced by a collapsing bubble is estimated directly from the numerical solution of the Rayleigh–Plesset equation (5). We assume that the nearest bubbles are located at a distance of the order of their instantaneous radius from the free surface; under this geometric condition the liquid velocity at the surface is taken to be approximately equal to the bubble-wall velocity d*R_b_*/d*t*. In the Weber criterion the sum

$v=\frac{d\xi }{\mathrm{dt}}+\frac{dR_{b}}{\mathrm{dt}}$ is formally retained, yet once inertial collapse develops the wall-velocity term dominates by one to two orders of magnitude. This estimate remains within the zero-dimensional coupled framework of the model and does not invoke any additional hydrodynamic solver.

In the present work the developed cavitation regime is identified quantitatively by the following indicators obtained from the simultaneous solution of Eqs. (2), (3), (4), (5):

- the occurrence of a rapid inertial contraction of the bubble (collapse), characterised by wall velocities |d*R_b_*/d*t*| that reach 1–10 km/s;
- radial oscillation amplitudes that become comparable to the thickness *h* of the deformed liquid disk;
- the liquid velocity entering the Weber criterion (11) being dominated by the bubble-wall velocity rather than by the capillary-wave crest velocity dξ/d*t*.

Thus, the primary destruction mechanism – capillary-wave or cavitation – will be determined by the ratio of the surface deformation rate. This will be determined either by the vertical component of the capillary wave velocity or by the wall velocity of the collapsing bubble located near the surface (whichever is higher).

The solution of equations (2), (5) are typical problems of nonlinear dynamics. Their solution is either stable, or catastrophic changes in the behavior of the dynamic system occur. Finding the conditions under which the system loses stability will allow us to determine the droplet fragmentation thresholds and the sizes of the resulting new droplets.

### 2.6 The size of new droplets taking into account the accumulation of energy in the oscillations of cavitation bubbles

Even if the scenario of bubble collapse inside a droplet and its significant influence on capillary waves does not materialize, bubble oscillations within the droplet will influence the size of new droplets due to the energy accumulated in these oscillations.

Taking cavitation processes into account, the size of new droplets can be estimated from the laws of conservation of energy and conservation of mass [13]. The idea is that cavitation bubbles accumulate ultrasonic energy during the time of exposure to droplet collapse (the induction time). If we denote the surface energy of a disk-shaped droplet as *E_drop_*, and the surface energy *n_d_* of new droplets of radius *R_new_* as *E_new_*, then *E_drop_ = n_d_ E_d1_*. From the law of conservation of mass, we obtain $n_{d}={\left(\frac{R_{d0}}{R_{\mathrm{new}}}\right)}^{3}$. Then, taking into account $E_{\mathrm{drop}}=2\sigma \pi R_{\mathrm{dc}}^{2}$, $E_{\mathrm{new}}=4\sigma \pi R_{\mathrm{new}}^{2},$ we obtain $R_{\mathrm{new}}=\frac{2R_{d0}^{3}}{R_{\mathrm{dc}}^{2}}.$ Note that during the time of destruction induction, the energy of the disc-drop becomes greater than just the surface energy – greater by the amount of accumulated ultrasonic energy in the oscillations of bubbles and capillary waves. If we take into account the accumulation of energy during oscillations of cavitation bubbles and capillary waves in the ultrasonic field *E_uz_*, then taking into account the conservation of the mass of the drop-disk and new drops, we obtain for the radius of new drops:

(12) $$R_{\mathrm{new}}\approx \frac{E_{drop0}R_{d0}}{E_{\mathrm{drop}}+E_{\mathrm{uz}}},$$

(13) $$E_{\mathrm{uz}}=\int_{0}^{t_{c}}\left(n_{b}E_{b}\left(t\right)+\sigma \xi^{2}\left(t\right)\right)dt.$$

This is the energy accumulated by all the bubbles in the droplet volume and the oscillating droplet surface during the fragmentation induction time *t_c_*, $E_{drop0}=4\sigma \pi R_{d0}^{2}.$ Here, $E_{b}\approx \frac{p_{d}^{2}}{c\rho }R_{0}^{2}t_{c}$ is the bubble energy,$n_{b}=\alpha {\left(\frac{R_{d0}}{R_{0}}\right)}^{3}.$

It should be noted that the energy accumulated by bubbles is several orders of magnitude greater than the energy accumulated by capillary wave oscillations. Therefore, the second term in equation (13) can be neglected, yielding:

(14) $$E_{\mathrm{uz}}=\frac{p_{d}^{2}}{c\rho }\int_{0}^{t_{c}}n_{b}R_{b}^{2}\left(t\right)dt\approx \frac{p_{d}^{2}}{c\rho }\frac{\alpha R_{d0}^{3}}{R_{0}}t_{c}.$$

From (12), it follows that the more ultrasonic energy a droplet accumulates, the smaller its fragmentation into droplets. The longer the fracture induction time, the more energy the disc-shaped droplet accumulates, according to equation (14).

In the capillary-wave fracture mechanism, expression (12) determines the radius of the formed droplets based on the amount of energy accumulated by the original droplet during the induction time. Its minimum size is limited by the value determined from the classical equation for capillary fracture of liquids (9), which yields the limiting (minimum) radius of new droplets in this problem. If the size of the formed droplets, calculated from expression (12), is smaller than the limiting value (9), then the value from expression (9) should be used as the radius of the formed droplets.

Thus, the developed model of capillary destruction of a levitating droplet allows one to determine the size of the formed droplets depending on the characteristics of the acoustic impact, the size and properties of the initial droplet, the number of cavitation bubbles, but does not allow one to overcome the physical limitations inherent in the capillary-wave detachment of droplets from the liquid surface (equation (9).

To form smaller droplets, it is advisable to implement a “cavitational” collapse mechanism (in the bubble collapse mode). In this case, the droplet size will be determined by expression (11), which includes the bubble wall collapse velocity parameter. This velocity is 1-2 orders of magnitude higher than the vertical velocity of the capillary wave crest in the capillary collapse scenario. According to formula (11), this leads to a sharp reduction in the size of the new droplets compared to the case of “quiet” capillary waves.

### 2.7 Threshold pressure levels for obtaining minimum droplet size in the capillary-wave destruction mechanism

To achieve the minimum droplet size upon breakup of the initial droplets, two conditions must be met: the excitation of parametric resonance on the droplet surface and the fulfillment of the droplet detachment condition according to the Weber criterion (8). Let's determine the threshold acoustic pressure required to achieve the maximum (minimum) droplet size. We obtain the first pressure threshold, which is determined from the following system of equations:

$$\left\{\begin{array}{c} R_{\mathrm{dc}}=R_{d0}+\frac{P_{cr1}^{2}}{4\rho_{g}c_{g}^{2}}\frac{R_{d0}^{2}}{\sigma }\\ R_{\mathrm{new}}=\frac{2R_{d0}^{3}}{R_{\mathrm{dc}}^{2}}=\frac{1}{k}={\left(\frac{4\sigma }{\omega^{2}\rho }\right)}^{1/3} \end{array}\right).$$

The first equation determines the size of the deformed droplet-disk, the second equation corresponds to the criterion for the droplet's separation from the wave crest, taking into account the laws of conservation of mass and energy. Hence:

(15) $$P_{cr1}\approx {\left[\frac{4\rho_{g}c_{g}^{2}\sigma }{R_{d0}}\left(\sqrt{\frac{2R_{d0}\omega^{2/3}}{{\left(4\sigma /\rho \right)}^{1/3}}}-1\right)\right]}^{1/2}.$$

The threshold pressure for achieving the minimum droplet size is determined by a complex system dependent on the initial droplet size, liquid properties (density and surface tension), and ultrasound frequency.

In addition to the threshold pressure for destruction, determined from physical considerations of droplet deformation and conservation laws (energy, mass), another threshold pressure must be determined – from the conditions for excitation of resonance on the droplet surface. From the condition for excitation of parametric resonance (equation (7)) $A_{\;\mathrm{mod}\;}>A_{\mathrm{cr}}=\frac{2\gamma_{v}}{\omega_{k}},$ it follows that: Then, it can be shown from the above expressions that

(16) $$P_{cr2}\approx \frac{8\eta \rho_{g}c_{g}^{2}}{\rho \omega }{\left(\frac{\omega^{2}\rho }{4\sigma }\right)}^{2/3}.$$

That is, the shorter the wavelength of ultrasonic vibrations, the greater the acoustic pressure required to excite parametric resonance. This explains why high-frequency ultrasound requires greater energies to excite capillary waves.

Equation (16) corresponds to the case where the disk-shaped droplet is not too thin relative to the wavelength: *hk* > 1. If the disk thickness *h* satisfies the condition *hk* << 1 (a thin disk, which is true under high-power ultrasonic vibrations, as in our case), we use the modified dispersion $k^{2}\approx \frac{\omega }{2}\sqrt{\frac{\rho }{\sigma h}}$ and obtain a system of equations for determining the critical pressure:

(17) $$\left\{\begin{array}{c} P_{cr2}\approx \frac{4\eta \rho_{g}c_{g}^{2}}{\sqrt{\rho \sigma h}}\\ h=\frac{4}{3}\frac{R_{d0}^{3}}{{\left(R_{d0}+\frac{P_{cr2}^{2}R_{d0}^{2}}{4\rho_{g}c_{g}^{2}\sigma }\right)}^{2}}. \end{array}\right)$$

In our case, when the droplet is subjected to significant deformation under high-power ultrasound, the critical pressure is independent of frequency but depends on the thickness of the droplet disk. This thickness, in turn, is determined by this pressure (the second equation in system (17)).

The threshold pressures determine the necessary and sufficient conditions for the breakup of the primary droplet into fragments of minimum size in an ultrasonic field. This requires excitation of resonance on the droplet surface and sufficient energy to achieve the minimum droplet size. The critical pressure for achieving the minimum droplet size will be the highest of Pcr1 and Pcr2. At acoustic pressures exceeding the critical pressure, the condition for excitation of capillary waves on the surface and breakup of the droplets, achieving the minimum size determined by equation (9), will be reached.

### 2.8 Cavitation excitation of capillary waves. Transition from capillary to cavitation droplet collapse

The mechanism of “quiet” capillary droplet collapse was discussed above. Cavitation bubbles within the droplet merely accumulate energy as a result of oscillations in the ultrasonic field, so that this energy is sufficient to form new droplets upon detachment from the capillary wave crests. In this mode, the bubbles do not collapse.

During advanced cavitation, it is the bubbles, not the external ultrasonic field, that drive the capillary waves. Below is the condition for the dominance of cavitation excitation of capillary waves.

Taking into account the influence of bubble pressure, based on the relationships presented above, we obtain:

$$p_{b}∼\rho n_{b}\frac{R_{0}}{r}R_{b}\frac{dR_{b}^{2}}{dt^{2}}∼\rho n_{b}k_{b}R_{0}\Delta R_{0}\omega^{2}=\rho \alpha \frac{R_{d0}^{3}}{R_{0}^{2}}\Delta R_{0}\sqrt[{3}]{\frac{3\alpha }{4\pi }}\omega^{2}∼\rho \alpha^{1.3}\frac{R_{d0}^{3}}{R_{0}^{2}}\Delta R_{0}\omega^{2}=\alpha^{1.3}\sigma \frac{R_{d0}^{3}}{R_{0}^{2}}\Delta R_{0}k^{3}.$$

Bubble wave excitation parameter: $A_{\;\mathrm{mod}\;b}∼\frac{p_{b}}{\sigma k}=\alpha^{4/3}\frac{R_{d0}^{3}}{R_{0}}k^{2}.$ If this parameter is greater than the ultrasound excitation, then we have: $\alpha^{4/3}\frac{R_{d0}^{3}}{R_{0}}\frac{\Delta R_{0}}{R_{0}}k^{2}⩾\frac{p_{\mathrm{ac}}}{c_{g}^{2}\rho_{g}}.$ This is the condition for the dominance of cavitation excitation of capillary waves. Here, Δ*R*_0_/*R*_0_ is the relative amplitude of the cavitation bubble oscillations, which, in turn, is proportional to the acoustic pressure *p_ac_*. Hence, the condition for the transition from ultrasonic to cavitation excitation is:

(18) $$\alpha_{\mathrm{cr}}⩾{\left(\frac{R_{0}}{c_{g}^{2}\rho_{g}k^{2}R_{d0}^{3}}\right)}^{3/4}.$$

Developed cavitation is identified in the numerical solutions by a rapid bubble collapse (wall velocities 1–10 km/s), oscillation amplitudes comparable to the disk thickness *h*, and dominance of the bubble-wall velocity in the Weber criterion (11). These features appear when α\alphaα exceeds the critical value given by Eq. (18). Thus, Eq. (11) replaces the capillary-based prediction (Eq. (9) precisely when α > α*_cr_*.

From the resulting expression, it follows that the transition to cavitation collapse mode occurs regardless of the acoustic pressure, but rather depends on the volume fraction of cavitation bubbles in the liquid. The threshold value α increases with increasing cavitation bubble size and decreases with droplet size and ultrasound frequency.

During the transition to cavitation mode, the vertical velocity of the capillary wave is no longer determined by its amplitude, but by the collapse velocity of the cavitation bubble v, which can reach 1-10 km/s and is determined from numerical calculations. The minimum size of new droplets is determined by formula (11).

To clarify the scope of the proposed fragmentation model and to summarize the physical effects incorporated into the formulation, a comparison with the conventional capillary-wave description is provided in Table 1. The comparison focuses on the main physical mechanisms relevant to ultrasonic droplet fragmentation, including acoustic-radiation-induced droplet deformation, cavitation-bubble dynamics and interactions, and the transition from capillary-wave to cavitation-induced breakup. It also indicates which additional capabilities, such as non-Newtonian rheology and more complex acoustic excitation, are possible within the general framework but have not been systematically investigated in the present study.

**Table 1:** Comparison of the main physical effects considered in the conventional capillary-wave description and in the present model.

Physical effect / capability	Conventional capillary-wave description	Present model
Capillary-wave instability and droplet detachment	Included	Included
Acoustic-radiation-induced droplet deformation	Usually simplified or prescribed	Explicitly included
Finite-thickness, deformed droplet geometry	Usually neglected	Included
Cavitation-bubble dynamics	Not explicitly considered	Included
Bubble-bubble interaction and collective dynamics	Not explicitly considered	Included
Bubble collapse and cavitation-induced breakup	Not explicitly considered	Included
Transition between capillary-wave and cavitation breakup	Not explicitly described	Explicitly investigated
Viscous effects	May be included depending on formulation	Included in the deformation model
Non-Newtonian rheology	Requires an appropriate constitutive formulation	Can be incorporated; not investigated in the present study
Complex / multi-frequency acoustic excitation	Usually formulated for prescribed excitation	Can be incorporated through a generalized acoustic forcing; not investigated in the present study
Breakup time and daughter-droplet size	Depending on model formulation	Calculated

As summarized in Table 1, the main distinction of the present formulation is the simultaneous consideration of acoustic deformation of the droplet, capillary-wave instability, and collective cavitation dynamics within the evolving droplet geometry. In particular, the model allows the change in the dominant fragmentation mechanism to be related to the development of cavitation and bubble collapse. The treatment of non-Newtonian rheology and complex acoustic excitation represents a possible extension of the framework and is not quantitatively assessed in the present study.

## 3 Results and discussion

### 3.1 Model testing. Identifying significant parameters

Numerical calculations were performed using equations (2-5). The mutual influence of cavitation bubble pressure in the Mathieu-Hill equation is accounted for using the *A_b_* factor in equation (3). Furthermore, the Weber criterion calculation takes into account the sum of the velocities $v=\frac{d\xi }{\mathrm{dt}}+\frac{dR_{b}}{\mathrm{dt}}$– the vertical velocity of the capillary wave crest and the velocity due to the motion of the bubble wall close to the surface. Characteristic curves of surface oscillations and bubble radius are shown in Fig. 2. The calculation is continued until the moment when the droplet collapses, ξ→∞.

**Fig. 2:**
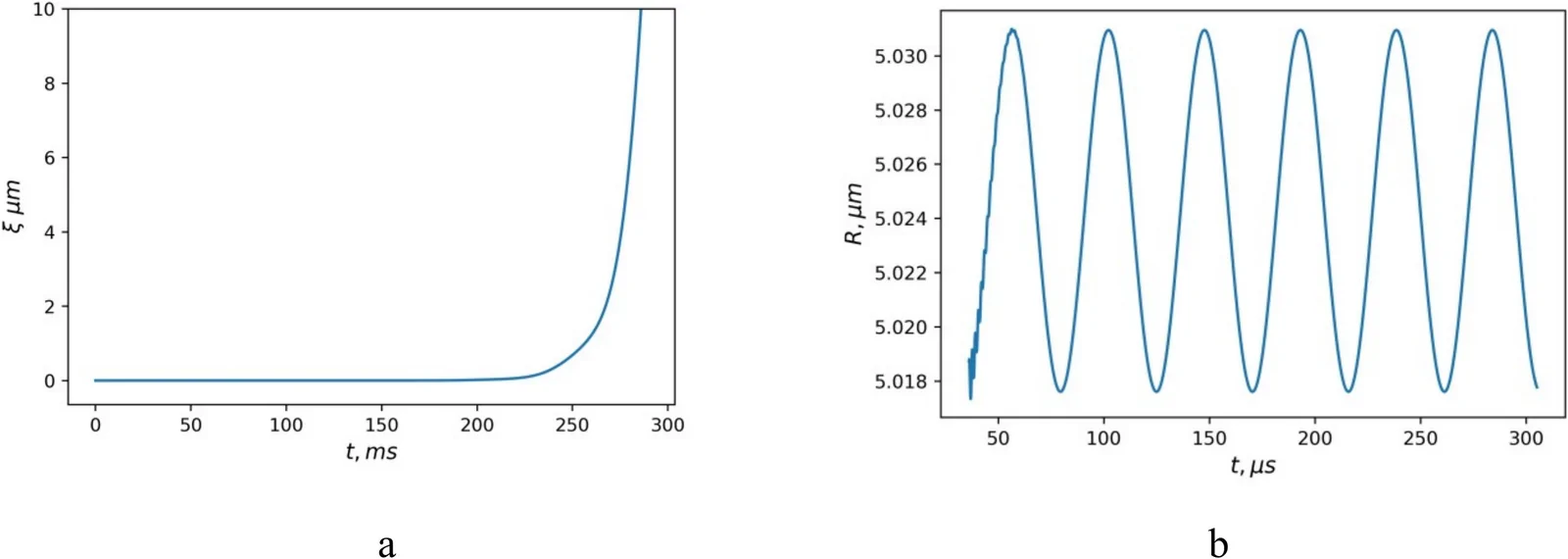
Oscillations of the surface (a) and cavitation bubble (b) in a levitating droplet. Calculated at *f* = 22 kHz, *p_ac_* = 63 kPa, *R_b_*_0_ = 5 μm, *R_d_*_0_ = 1 mm. Bubble interaction is ignored (*p_b _*= 0 in equation (5)).

A droplet breakup scenario is realized as a result of capillary wave instability (a sharp increase in the surface wave, Fig. 2a). In this case, the cavitation bubbles do not collapse, but merely oscillate with a small amplitude. A numerical calculation yields a droplet diameter of 24.4 μm according to the Weber criterion (8), while an analytical estimate (9) yields 24.7 μm (the numerical and analytical calculations thus yield approximately the same result). Thus, the analytical model in this case adequately describes the droplet breakup process. The breakup time is 290 ms.

If the influence of the pressure of neighboring bubbles *p_b_* is taken into account in the bubble oscillation equation (5), breakup (as a result of capillary instability) in this calculation occurs in a shorter time (244 ms) (Fig. 3).

**Fig. 3:**
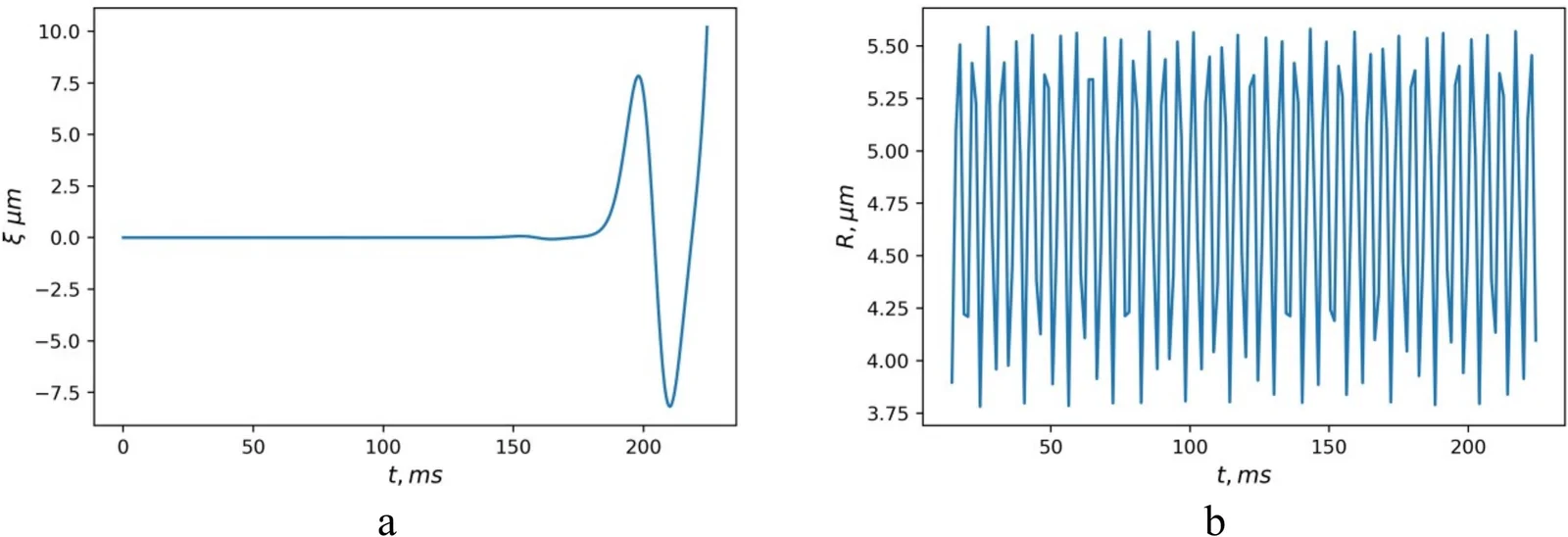
Oscillations of the surface (a) and cavitation bubble (b) in a levitating drop. Calculation at *f* = 22 kHz, *p_ac_* = 63 kPa, α = 0.15, ω*_k_* = ω / 2, *R_b_*_0_ = 5 μm, *R_d_*_0_ = 1 mm, taking into account the mutual pressure of the bubbles (*p_b_* is not equal to 0 in equation (5)).

If the parameter values in the calculation are such that bubble collapse does not occur, droplet breakup occurs as a result of capillary wave instability. The size of the new droplets in the numerical calculation corresponds to the analytical estimate (9), and the mutual pressure influence of neighboring bubbles has little effect on the process dynamics (slightly reducing the breakup time, but the size of the new droplets remains the same). A different picture occurs if the bubbles in the liquid droplet collapse (the mechanism of developed cavitation).

As discussed in Section 2.6, the occurrence of a particular breakup scenario (due to capillary wave instability or bubble collapse) depends significantly on the bubble concentration in the liquid. The higher the concentration, the higher the probability of droplet breakup via bubble collapse (the cavitation mechanism). In the model, this is primarily determined by the factor kb, which is equal to the ratio of the initial size of the cavitation bubble to the characteristic distance between the bubbles. The scenario of destruction due to instability and bubble collapse is realized in the calculation (all other things being equal) taking into account the pressure from neighboring bubbles and with a volume fraction of cavitation bubbles α > 0.377 (Fig. 4). In this case, the destruction time of the initial levitating droplet is sharply reduced (11 ms in this calculation), and the size of the new droplets becomes ∼1 μm.

**Fig. 4:**
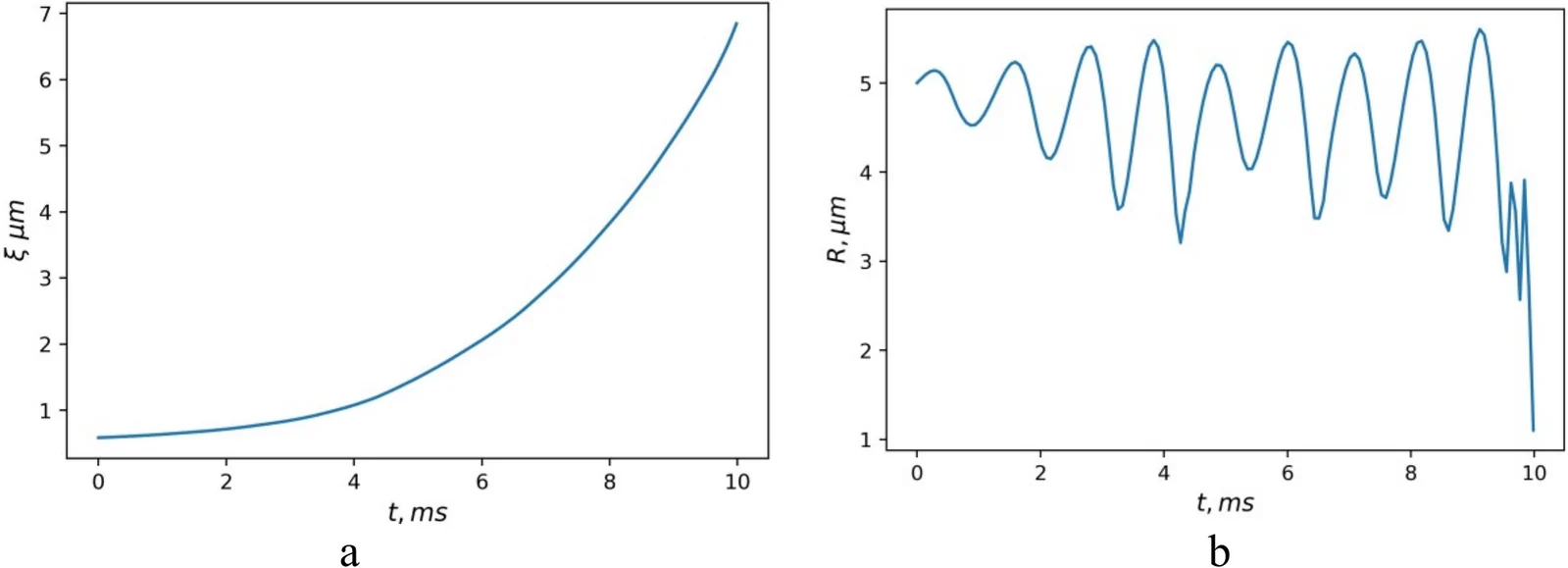
Oscillations of the surface (a) and cavitation bubble (b) in a levitating drop. Calculation at *f* = 22 kHz, *p_ac_* = 63 kPa, ω*_k_* = ω / 2 (resonant frequency of capillary waves), *R_b_*_0_ = 5 μm, *R_d_*_0 _= 1 mm, α = 0.38.

Important factors influencing the breakup mechanism, droplet fragmentation time, and the size of new droplets include pressure and frequency of impact, the volume fraction of bubbles (gas saturation of the liquid), their characteristic size, the size of the primary droplet, and the viscosity and surface tension of the liquid. We will conduct a parametric study of the model.

### 3.2 Parametric study of the model

#### 3.2.1 Effect of acoustic pressure amplitude and energy limitations

The following conditions were adopted for the numerical calculations: α = 0.15, *f* = 22 kHz, *R*_0_ = 5 μm, *R_d_*_0_ = 1 mm and 5 mm. The acoustic pressure amplitude varied from 5 to 63 kPa. The destruction mechanism is the breakdown of the capillary wave stability. The destruction time depends on the acoustic pressure, increasing from approximately 50 ms at *P_ac_* = 5 kPa to approximately 280 ms at *P_ac_* 63 kPa. The average destruction time over the investigated pressure range is approximately 170 ms. The minimum diameter of new droplets, according to expression (9), is 24.7 μm. The sizes of new droplets, calculated in accordance with the “energy” calculation, expression (12), are shown in Fig. 5.

**Fig. 5:**
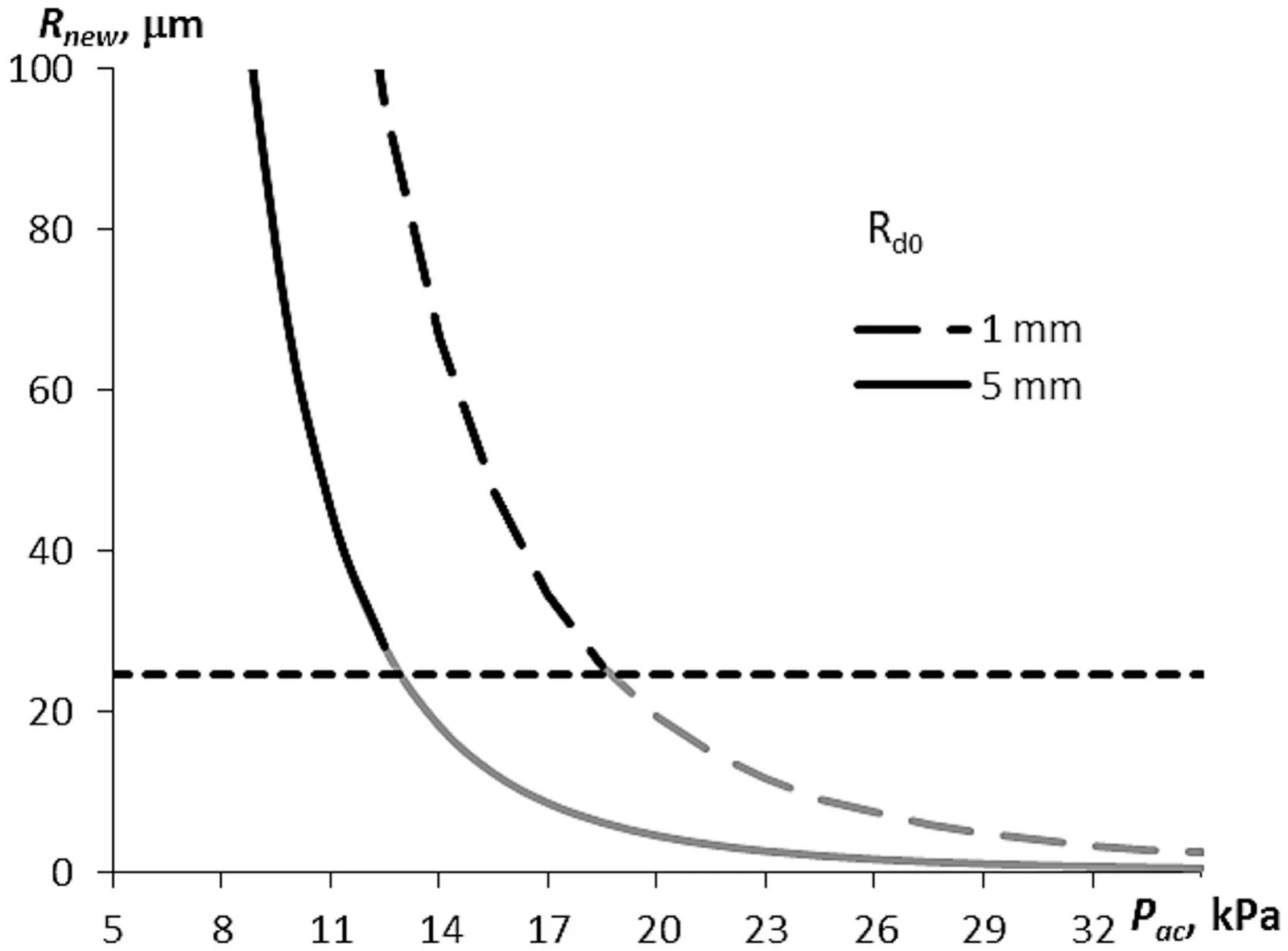
Dependence of the size of new droplets on the acoustic pressure (α = 0.15, *f* = 22 kHz, *R*_0 _= 5 μm, *R_d_*_0_ = 1 mm and 5 mm; the dashed line corresponds to the minimum limit size according to the capillary model (9), 24.7 μm).

To assess the robustness of the calculated droplet size with respect to the parameters describing cavitation dynamics, additional calculations were performed by varying the initial cavitation nucleus radius *R*_0 _and the pressure-transmission coefficient *k_T_*. The reference calculations were performed with *R*_0 _= 5 μm. Variation of *R*_0 _within 1–10 μm changes the calculated *R_new_* by no more than approximately 1–2%. Variation of *k_T_*, including the coefficient *k_T_*_1_ in Eq. (6), produces an even smaller change in *R_new_*, not exceeding approximately 1% (Table2). Thus, the predicted droplet size is weakly sensitive to these parameters, and the main trends obtained from the parametric calculations are robust with respect to their variation. Neither *R*_0_ nor *k_T_* was adjusted to reproduce the experimental droplet sizes.

**Table 2:** Sensitivity of the predicted new droplet radius to variations in model parameters.

Model parameter	Reference value	Tested range	Maximum change in *R_new_*
*R*_0_	5 μm	1–10 μm	1–2 %
*k_T_*_1_	270 m^−1^	0–2700 m^−1^	< 1 %

The droplet diameter corresponding to that calculated from the capillary model will be achieved at *p_ac_* > 18.7 kPa for a droplet with a radius of 1 mm, and 12.9 kPa for a droplet with a radius of 5 mm. If the pressure is lower than 18.7 or 12.9 kPa in these calculations, the initial droplet will not have enough energy to achieve the minimum droplet size of 24.7 μm, and the resulting droplets will be larger than this minimum size.

A further increase in acoustic pressure allows the droplet to accumulate more ultrasonic energy during the induction period, but will not reduce the droplet size below the limiting size (classical equation (9)), derived from the capillary breakage model.

However, if droplet breakage occurs as a result of bubble collapse (cavitation breakage mechanism), the size of the new droplets will not be limited by the size derived from the capillary mechanism (24.7 µm in this calculation), but will be determined by equation (11). That is, the limiting size in the cavitation regime during bubble collapse is not determined by equation (9) but will be smaller.

As follows from formula (9), the minimum size of new droplets depends on the frequency of impact, density, and surface tension of the liquid. Calculations for water show that the minimum radius of new droplets decreases almost linearly from 29 µm at *f* = 17 kHz to 19 µm at *f *= 35 kHz [13].

#### 3.2.2 Threshold pressure for achieving minimum droplet size

This pressure is defined as the maximum of two threshold values, *P_cr_*_1_ (15) and *P_cr_*_2_ (equations (16) or (17)). The former determines the minimum pressure that allows new droplets to be formed from the initial droplet while maintaining energy and mass. The latter determines the minimum pressure required to excite oscillations of the droplet surface, thereby enabling its destruction. In this case, the droplet will break up into the smallest possible fragments, the size of which depends on the frequency of the impact and the properties of the liquid.

As calculations show, for small initial droplets, for which the condition *kh* << 1 is satisfied during deformation, *P_cr_*_2_, calculated using equation system (17), will be the determining factor. For larger initial droplets, for which *kh* >> 1, *P_cr_*_2_ will also be the determining factor, but calculated using formula (16). In the intermediate range of droplet sizes, the pressure *P_cr_*_1_ plays a role (Fig. 6a).

**Fig. 6:**
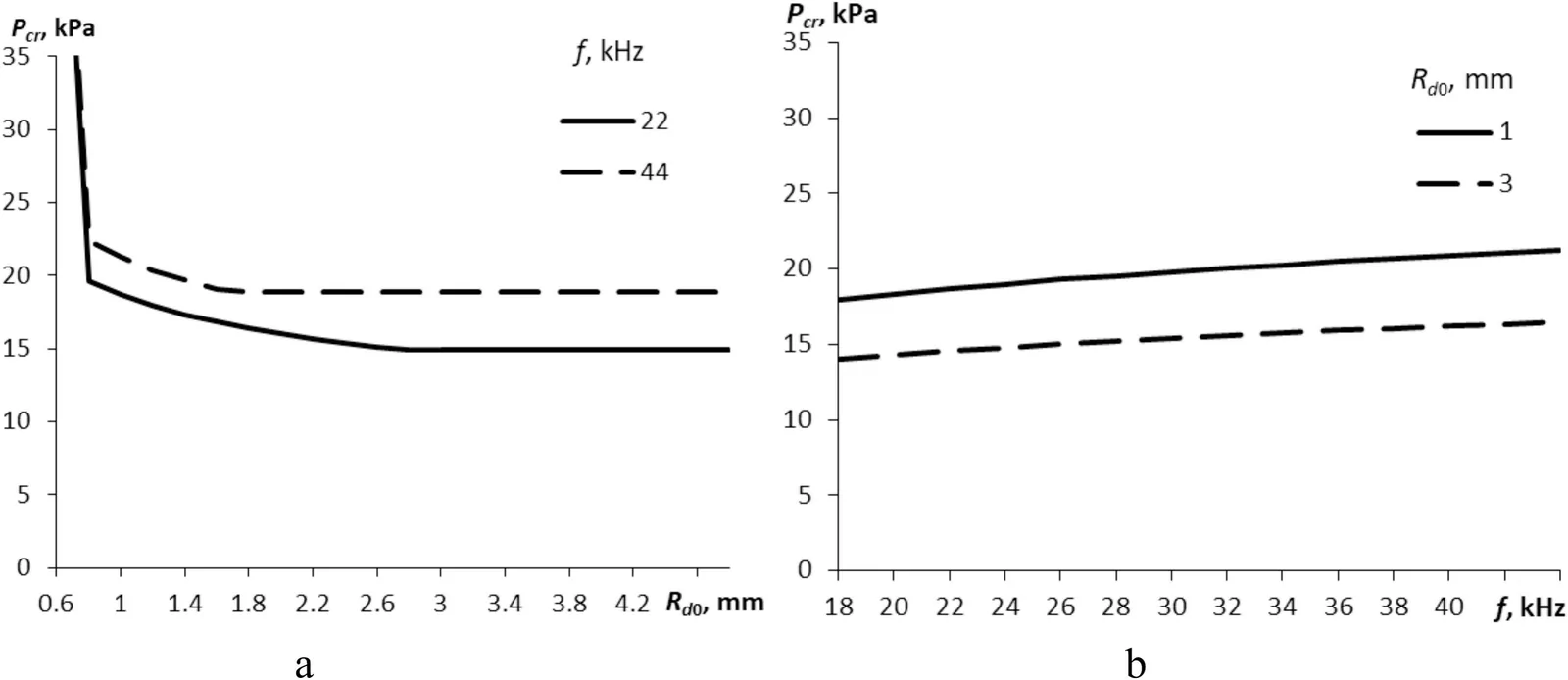
Dependence of the critical level of acoustic pressure required to break up a primary droplet to achieve the minimum size of new drops on the size of the primary droplet (a) and on the frequency (b).

Thus, under the condition *hk* is greater than or of the order of 1, the critical pressure for excitation of a capillary wave is determined by equation (16) and is independent of the droplet size, but depends on the oscillation frequency (increases with increasing frequency, Fig. 6b). Under the condition *hk* << 1, which in our problem is realized only for very small primary droplets – less than 0.5 mm – the system of equations (17) is applied. In this case, the critical pressure depends significantly on the droplet radius, and the smaller the droplet, the greater the pressure required to excite capillary waves. However, as can be seen from the system of equations (17), in this case this pressure is independent of the oscillation frequency. For example, for a water droplet with a radius of 0.5 mm, an acoustic pressure of 84 kPa is required to excite capillary waves, regardless of the excitation frequency. And for a droplet with a radius of 2.8 mm, the critical pressure level will be only 15 kPa at a frequency of 22 kHz and 19 kPa at a frequency of 44 kHz.

#### 3.2.3 Effect of gas content in liquid. Transition from capillary-wave to cavitation droplet destruction mechanism

As shown above, in the capillary-wave breakup mode, when cavitation bubbles do not collapse inside the droplet but merely oscillate, the ultimate minimum size of new droplets will be the one determined by classical equation (9). However, in the cavitation mode, when bubbles collapse and disrupt the droplet surface with sharp pulses, the sizes of new droplets will be smaller (determined by equation (11). Let us consider the conditions for the transition from the capillary to the cavitation mode of droplet breakup.

As follows from equation (18), a change in the gas content of the liquid leads to a change in the droplet breakup mode from capillary instability to bubble collapse. Fig. 7a shows the calculated values of the droplet breakup time (numerical calculation at *p_ac_* = 35,000 Pa, *f *= 22 kHz, *R*_0_ = 5 μm, *R_d_*_0_ = 1 mm).

**Fig. 7:**
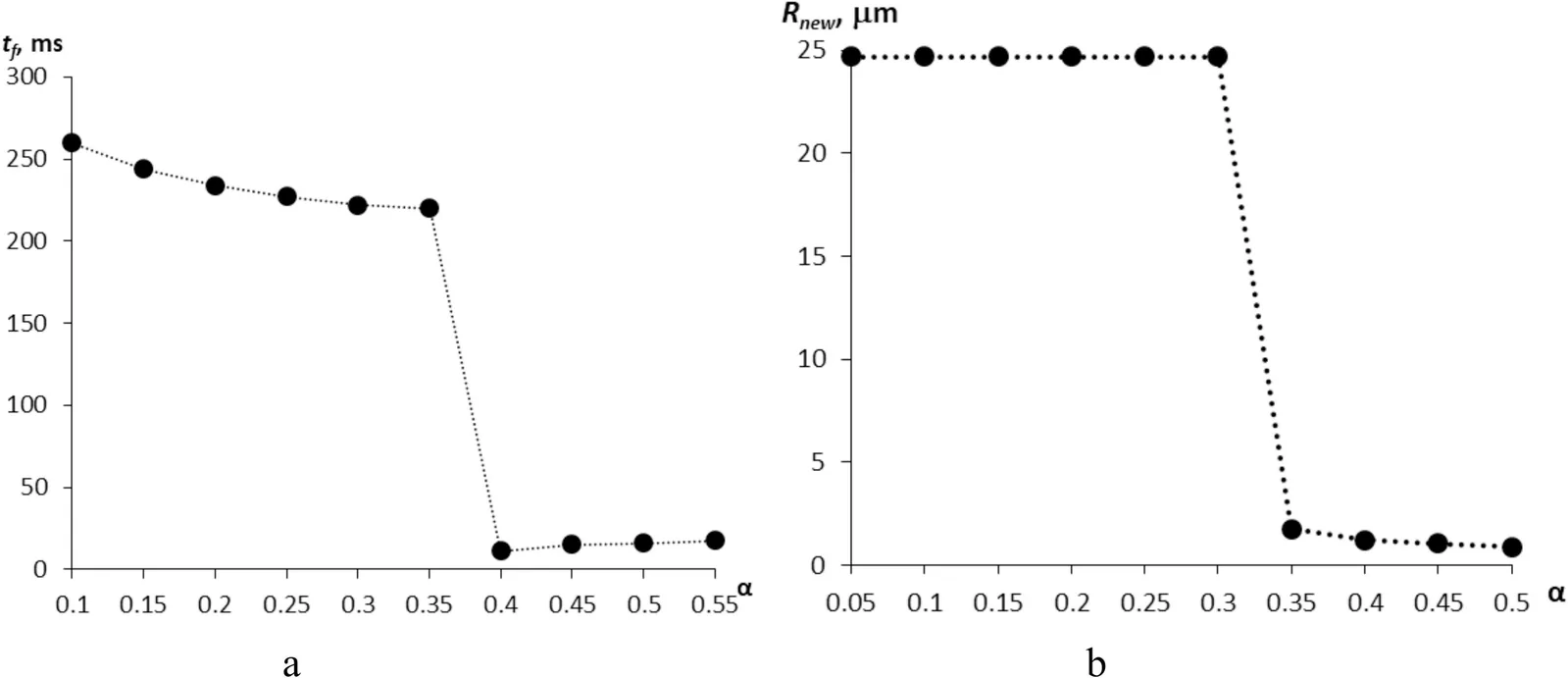
Dependence of fragmentation time (a) and size of new droplets (b) on the volume fraction of cavitation bubbles in the liquid calculations (calculation at *p_ac_* = 35000 Pa, *f* = 22 kHz, *R*_0 _= 5 μm, *R_d_*_0_ = 1 mm).

From Fig. 7a, it follows that at α < 0.1, droplet breakup does not occur (there is insufficient energy to cause capillary instability or bubble collapse). At α > 0.35, a transition from capillary droplet breakup to cavitation (from bubble collapse) occurs. This change corresponds to the onset of the developed cavitation regime defined by the quantitative indicators listed in Section 2.5 (rapid bubble collapse with wall velocities of 1–10 km/s and oscillation amplitudes comparable to the disk thickness *h*). The physical interpretation is as follows: as the volume fraction of bubbles increases, their influence on the rate of capillary instability development increases, due to the bubbles' proximity to the surface and the pressure pulses generated by their oscillations. As the volume fraction of bubbles continues to increase, their mutual influence also increases, leading to instability of bubble oscillations and bubble collapse. Moreover, this occurs faster than capillary wave instability could develop.

Fig. 7b shows the calculated values of the new droplet sizes (calculated using analytical formulas (9) and (11), taking into account the breakup times obtained in the numerical calculation). When the capillary destruction mechanism is realized, the minimum size of new droplets is determined by formula (9), and the energy accumulated during the induction period is sufficient to form such droplets. When switching to the cavitation destruction mechanism at α > 0.35, the droplet size is not limited from above by expression (9), since the vertical velocity in the surface wave during bubble collapse is orders of magnitude greater than that realized during resonant instability of the capillary wave (equation (11)). Thus, during the transition to cavitation destruction, the droplet fragments not only an order of magnitude faster than during capillary-wave destruction (10-15 s instead of 260-220 s), but the size of the new droplets also becomes an order of magnitude smaller (approximately 1 μm instead of 25 μm).

Thus, the gas content (gas volume fraction α) turned out to be a critical parameter determining which of the two mechanisms breaks the droplet first. Three distinct regions are identified:

1. α < 0.1 – no breakup;
2. α ∼ 0.1 – 0.35 – capillary breakup mechanism, breakup time ∼250–300 ms, new droplet size ∼25 μm (formula (9));
3. α > 0.35 – cavitation breakup mechanism, breakup time ∼10–15 ms, new droplet size ∼1 μm (formula (11)).

As the bubble volume fraction increases, two sequential effects occur:

1. Strengthening of capillary waves. Bubbles near the surface create local pressure pulses (Equation (4)). These pulses further oscillate the surface, accelerating the growth of capillary instability. However, up to α ≈ 0.35, this effect does not change the physics of the process: the droplet still breaks up due to detachment from the wave crests.
2. Collective collapse of bubbles. When α exceeds ∼0.35, the distance between bubbles becomes so small that their mutual pressure (*p_b_* in Equation (5)) causes instability of radial oscillations. The bubbles begin to collapse, generating shock waves and microjets. This process develops faster than capillary instability (Fig. 7a: the time drops from 250 to 10 ms). The destruction mechanism changes qualitatively: now it is not waves, but collapsing bubbles that “knock out” the liquid from the surface.

Why does droplet size decrease sharply upon passing the critical value of α (Fig. 7b)? In the capillary-wave mechanism, the minimum size of new droplets is determined by the classical formula (9). For water at a frequency of 22 kHz, this is ∼25 μm. The ultrasound energy accumulated during the induction time (250 ms) is sufficient to “pay for” the surface energy of such droplets.

In the cavitation regime, the situation is different. The liquid velocity initiated by bubble collapse is 2–3 orders of magnitude higher than the capillary wave crest velocity. Substituting this velocity into the Weber criterion (equation (11)), yields droplet sizes in the micron and submicron range.

Thus, the transition from capillary to cavitation fragmentation is not a smooth change, but a bifurcation: for α > 0.35, the fragmentation time decreases by an order of magnitude, and droplet size also by an order of magnitude. This opens the possibility of controlling aerosol dispersion by controlling the gas content of the liquid.

As calculations have shown, the transition threshold from capillary to cavitation (bubble collapse) does not depend on acoustic pressure, but only on the bubble volume fraction, and occurs in the calculation at *f* = 22 kHz, *R*_0_ = 5 μm, *R_d_*_0_= 1 mm, and α*_cr_* ∼ 0.375. With such a dense distribution of bubbles in the liquid, they influence each other, leading to instability. The threshold values of αcr, calculated according to equation (18), are shown in Fig. 8.

**Fig.8:**
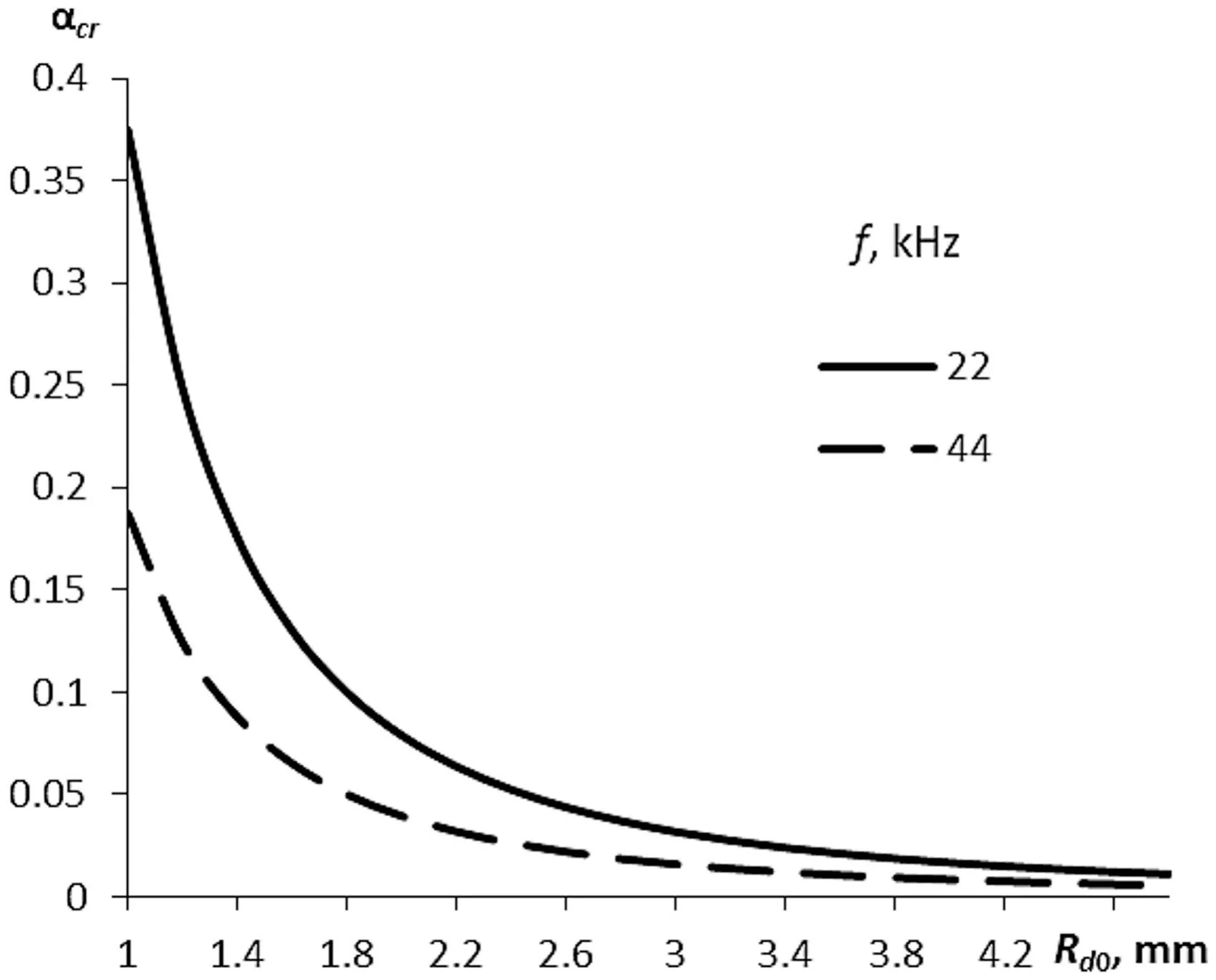
Threshold values of the volume fraction of cavitation bubbles for the transition from capillary instability to bubble collapse. Calculated for *R*_0_ = 5 µm.

As droplet size increases, the critical value of α decreases: for larger droplets, cavitation breakup occurs even at low gas saturation values, and for droplet radii less than 1 mm, it does not occur at all. Increasing the frequency of impact lowers this threshold. Breakup time becomes an order of magnitude shorter, and the size of new droplets also decreases by an order of magnitude. Thus, larger droplets will break up significantly faster and into smaller fragments than smaller primary droplets, all other things being equal.

#### 3.2.4 Effect of surface tension

As already noted, surface tension has a minor effect on the transition threshold from capillary-wave breakage to cavitation breakage, resulting in a reduction in breakage time. The purer the liquid, the longer it will take for its droplets to break apart, while a gas-saturated or inhomogeneously contaminated liquid will break apart an order of magnitude faster and into smaller droplets. Surface tension has a somewhat more significant effect on the size of new droplets in the capillary-wave breakage mechanism (Fig. 9). As surface tension increases, the size of the new droplets increases.

**Fig. 9:**
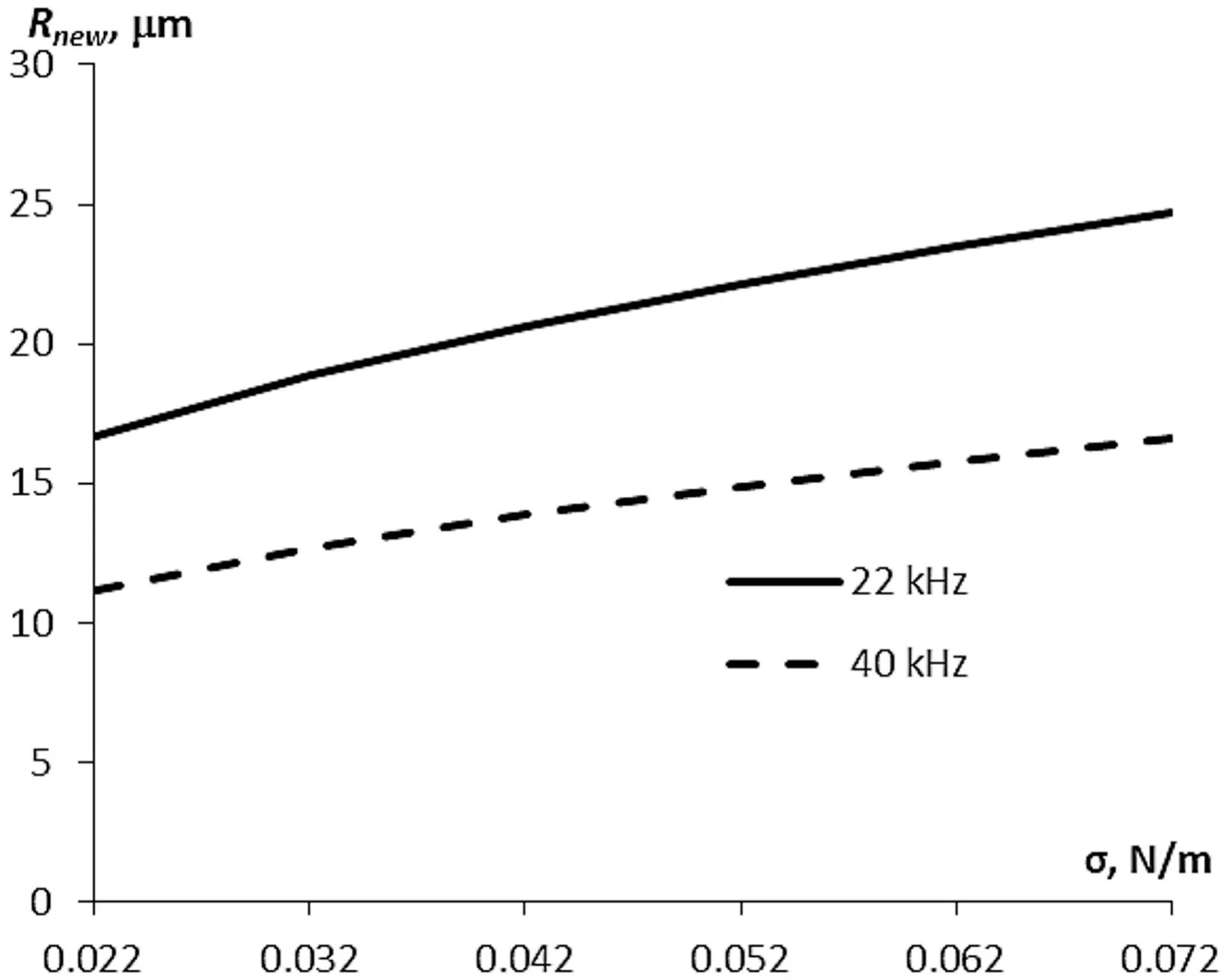
Minimum size of new droplets during capillary-wave destruction (*p_ac_* = 63 kPa, *R*_0_ = 5 µm, *R_d_*_0_ = 1 mm).

The calculated values of the radius of new drops for water, glycerin and alcohol correspond to the experiment [13]: 18, 22 and 28, respectively, for a frequency of 22 kHz.

It should be noted that throughout the model the surface tension σ is treated as a constant (flat-interface) value. A thermodynamic curvature correction of the Tolman type, σ(*R*) ≈ σ_∞_(1 − 2δ / R), exists in principle. For the parent droplets (millimetre scale) and for the capillary-regime daughter droplets (∼20–28 µm) the relative correction is of order 10^-5^–10^-4^ and is negligible. Even for the smallest droplets predicted in the cavitation regime (∼1 µm) and for the bubble radii attained in the numerical trajectories of Fig. 2, Fig. 3, Fig. 4, a conservative estimate with δ = 0.1nm yields δ / R ≲ 10^−3^. The resulting change in σ remains well below other uncertainties of the model (pressure transmission coefficient, inter-bubble coupling, etc.). Consequently, the constant-σ approximation is retained; the possible influence of curvature corrections for substantially smaller nuclei or more extreme collapse conditions is left for future refinement.

## 4 Conclusion

In this paper, we propose a comprehensive, coupled physical and mathematical model for the secondary fragmentation of levitating liquid droplets in a powerful ultrasonic field. The model's novelty lies in its combined consideration of droplet deformation under radiation pressure, parametric excitation of capillary waves, the radial dynamics of cavitation bubbles with their collective interactions, and energy accumulation in the system. This approach allowed us, for the first time, to describe interconnected, rather than isolated, fracture mechanisms within a single problem formulation.

For the first time, quantitative criteria for the transition between the two fragmentation regimes were identified. It was shown that the determining parameter is not only the acoustic pressure but also the volume fraction of gas α in the liquid. It was found that fragmentation does not occur at α < 0.1. In the range of 0.1 < α < 0.35, the capillary-wave mechanism dominates, with fragmentation times of ∼250–300 ms and the minimum droplet size determined by the classical relationship for water at a frequency of 22 kHz being ∼ 24.7 μm. When the threshold value α_cr_ ∼ 0.35–0.38 is exceeded, a sharp transition to the cavitation mechanism of bubble collapse occurs. In this regime, the fragmentation time is reduced by an order of magnitude to ∼10–15 ms, and the resulting droplet size is also reduced by an order of magnitude to ∼1 μm, as confirmed by numerical calculations using the Weber criterion, taking into account the bubble wall collapse velocity.

Analytical relationships for the threshold pressures required to achieve the minimum droplet size are established. It was shown that for a droplet with a radius of 1 mm at a frequency of 22 kHz, the threshold pressure is ∼18.7 kPa, and for a droplet with a radius of 5 mm, it is ∼12.9 kPa. Moreover, for thin disks (*kh* << 1, realized for droplets smaller than 0.5 mm), the critical pressure for excitation of capillary waves is independent of frequency and can reach 84 kPa for a droplet with a radius of 0.5 mm, whereas for larger droplets (e.g., 2.8 mm), it is only 15–19 kPa in the frequency range of 22–44 kHz. The calculated values ​​of the radii of new droplets for water (28 μm), glycerol (22 μm), and alcohol (18 μm) at a pressure of 35 kPa and a frequency of 22 kHz are in quantitative agreement with the experimental data, confirming the adequacy of the proposed model. Thus, the developed model allows for the prediction of aerosol fragmentation modes and dispersion, as well as targeted control of these processes by monitoring the liquid's gas content, the initial droplet size, and the ultrasonic parameters. This opens the door to the development of energy-efficient, contactless spraying technologies with tailored characteristics.

## CRediT authorship contribution statement

**Olga Kudryashova:** Writing – original draft, Conceptualization. **Andrey Shalunov:** Writing – review & editing, Supervision, Resources, Project administration, Funding acquisition, Conceptualization. **Roman Dorovskikh:** Methodology. **Sergey Titov:** Methodology, Investigation. **Natalia Titova:** Investigation.

## Funding

The study was supported by grant No. 23-19-00875-П from the Russian Science Foundation, https://rscf.ru/project/23-19-00875-П

## Declaration of competing interest

The authors declare that they have no known competing financial interests or personal relationships that could have appeared to influence the work reported in this paper.
